# Establishing a major cause of discrepancy in the calibration of Affymetrix GeneChips

**DOI:** 10.1186/1471-2105-8-195

**Published:** 2007-06-11

**Authors:** Andrew P Harrison, Caroline E Johnston, Christine A Orengo

**Affiliations:** 1 Department of Biological Sciences, University of Essex, Wivenhoe Park, Colchester, Essex, CO4 3SQ, UK; 2 Department of Mathematical Sciences, University of Essex, Wivenhoe Park, Colchester, Essex, CO4 3SQ, UK; 3 Department of Biochemistry, University College London, Gower Street, London, WC1E 6BT, UK

## Abstract

**Background:**

Affymetrix GeneChips are a popular platform for performing whole-genome experiments on the transcriptome. There are a range of different calibration steps, and users are presented with choices of different background subtractions, normalisations and expression measures. We wished to establish which of the calibration steps resulted in the biggest uncertainty in the sets of genes reported to be differentially expressed.

**Results:**

Our results indicate that the sets of genes identified as being most significantly differentially expressed, as estimated by the z-score of fold change, is relatively insensitive to the choice of background subtraction and normalisation. However, the contents of the gene list are most sensitive to the choice of expression measure. This is irrespective of whether the experiment uses a rat, mouse or human chip and whether the chip definition is made using probe mappings from Unigene, RefSeq, Entrez Gene or the original Affymetrix definitions. It is also irrespective of whether both Present and Absent, or just Present, Calls from the MAS5 algorithm are used to filter genelists, and this conclusion holds for genes of differing intensities. We also reach the same conclusion after assigning genes to be differentially expressed using t-statistics, although this approach results in a large amount of false positives in the sets of genes identified due to the small numbers of replicates typically used in microarray experiments.

**Conclusion:**

The major calibration uncertainty that biologists need to consider when analysing Affymetrix data is how their multiple probe values are condensed into one expression measure.

## Background

Microarrays provide the opportunity to study the transcriptional output of a genome. The most common application of microarrays at present is to perform comparative studies, looking for relative changes between two conditions. The aim of calibration is to minimise as much systematic and experimental variation in the data, whilst retaining the biological variation. The calibration of microarrays, combined with statistics of the changes, underpins what can be inferred from these studies.

In this paper we focus on the calibration of oligonucleotide GeneChip arrays produced by Affymetrix. The calibration process for GeneChips can be considered to have three parts: background correction; normalisation; expression measure. Background correction is needed to remove the proportion of intensity that is not due to hybridisation of the target. The normalisation of an array is needed before it can be compared against other arrays, correcting for differences in overall mRNA levels and scanner settings. An expression measure is needed to transform the fluorescence from an array into a measure of the mRNA abundance.

The aim of differential expression statistics is to highlight which genes show the most significant changes between two conditions. Z-scores can be used to measure the significance of a gene's fold change with respect to the population of changes seen elsewhere on the array [1]. The use of t-statistics is also commonly used to report genes that are differentially expressed. Throughout this work we refer to background correction, normalisation and expression measure as calibration and assessment of differential expression as statistics.

The preprocessing of GeneChips is an active research field with a number of different algorithms being developed [2]. This has led to comparative studies of the differences between different protocols ([2], [3]), with [4] noting the importance of background correction. Other work has compared the relative importance of differential statistics and normalisation and concluded that normalisation was the limiting factor in determining a set of genes [5]. However, it has been reported [2] that calibration methods that differ only in normalization result in practically identical expression measures. A full calibration includes several other steps as well as normalisation, and is usually followed by estimations of differential expression between two conditions. Given the uncertainty in the literature we therefore chose to study all the calibration steps in order to shed light on which of normalization, background correction or expression measure has the biggest effect on the sets of genes reported to be differentially expressed. We also wished to determine whether our conclusions about the dominant step depended on the choice of statistics used to infer differential expression.

A further complication is that some of the probes in a probeset may map to more than one transcript, and may not even map to the transcript of interest [6]. Moreover, information about probe mapping can be derived from several online resources, and not all of these will be in agreement [7]. A further complication with mapping is that some probes map to transcripts that undergo alternative splicing and alternative polyadenylation [8].

A full calibration protocol can be devised from one of the choices for each of the various strategies for normalisation, background correction and expression measures. Each of these protocols will produce a list of genes that are measured to be significantly differentially expressed. However, different protocols can produce different lists and the end-user biologist may worry that the wrong choice of protocol is producing misleading conclusions. We have sought to identify the calibration step which results in the biggest cause of change.

## Results

### 1) Sensitivity of different calibration protocols

The package affy allows a choice for each of the three primary calibration steps: three options for background correction; five choices for normalisation; three different expression summaries. In total, there are 45 different permutations for calibration (Table 1). After data has been calibrated, a comparison between two conditions allows genes to be identified as differentially expressed. We have used the Z-score method [1] as our statistical measure, and have collated the genes whose absolute measure of Z is the largest, i.e. we produce one list that includes both and up down regulated genes.

**Table 1:** Calibration options within Affy

Calibration step	Option 1	Option 2	Option 3	Option 4	Option 5
Background subtraction	Nothing	RMA	MAS		
Normalisation	Constant	Invariant set	Lowess	Qspline	Quantiles
Expression Measure	Li-Wong (MBEI)	Median Polish (RMA)	MAS5		

In order to assess the agreements in gene lists identified between different protocols we explored various strategies. We ultimately decided to compare the total amount of genes common to two lists as being our measure of consensus. For such a comparison we do not care if gene X is found to be the most significantly changing gene for protocol A and is found to be the 100^th ^most significantly changing gene for protocol B, just as long as gene X is found somewhere in both lists.

In order to provide a visual aid in assessing consensus between different protocols we have transformed percent overlap onto a greyscale: 0% overlap between two lists is coloured black, 100% overlap (the list members are identical) is coloured white and in between 0 and 100 are different shades of grey. The resulting matrix and corresponding overlap image is diagonally symmetric.

**Figure 1:**
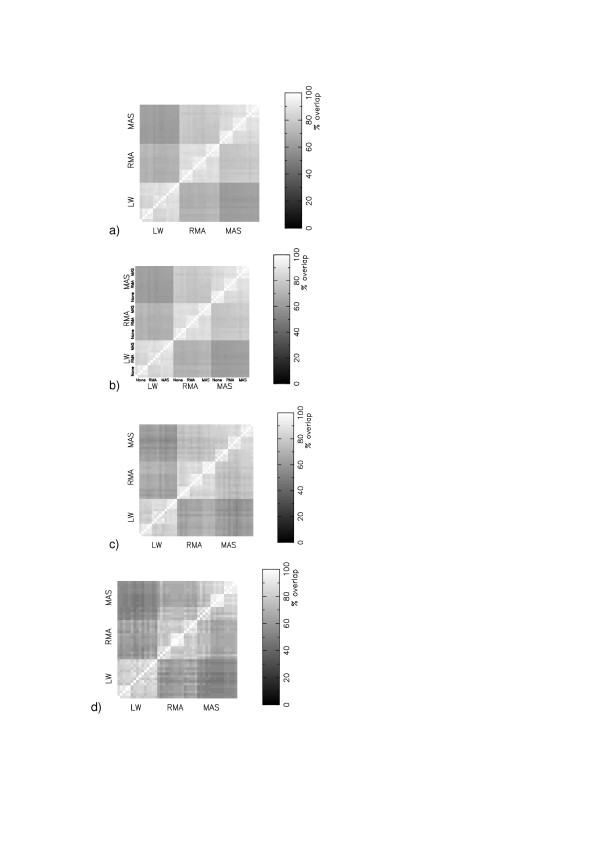
Matrices of consensus in the most significant changing genes (Z-score) in the 45 different protocols for GSE1004, for different cutoffs: (a) Top 400 genes. The blocks on consensus are labelled LW, RMA and MAS to indicate the choice of expression measure.; (b) 400 genes. We have labelled the next level of structure None, RMA and MAS, and this level indicates the choice of background subtraction.; (c) Top 100 genes ; (d) Top 30 genes

Figure 1 shows how the agreement between different protocols, for dataset GSE1004 [9], becomes increasingly noisy as we reduce the size of the gene lists that are compared from 400, to 100, to 30. Given the smoothness apparent for a list size of 400 genes we kept with this size for all subsequent comparisons. Figure 1 shows that the most significant clustering of agreement is that between different protocols which all have the same expression measure, whether that be RMA [10], MAS5 [11] or Li-Wong [12]. Two protocols that differ in background subtraction and normalisation, but have a common expression measure, will share more significant genes in common than two protocols that differ in expression measure, but which have a either a common background subtraction or normalisation or both.

**Figure 2:**
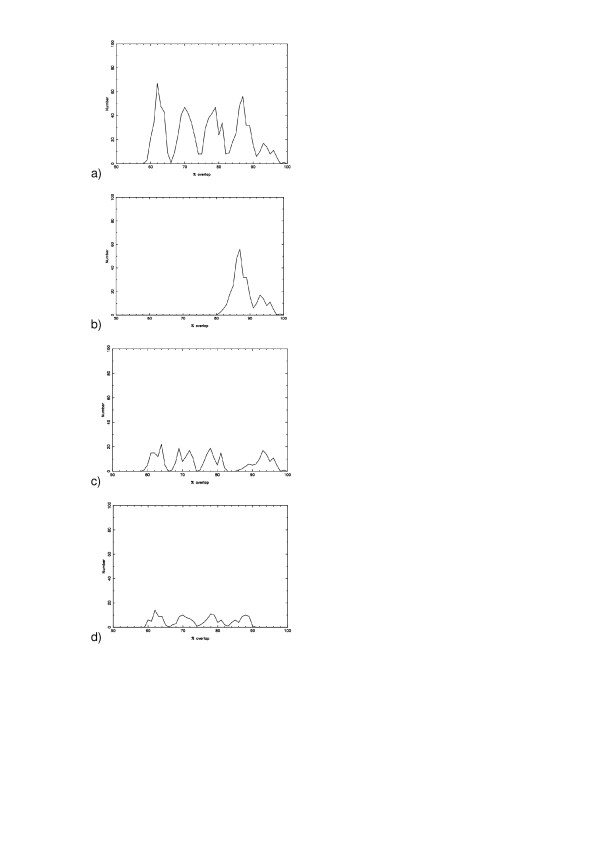
A histogram of the percent overlaps between two gene lists, produced from: (a) all permutation pairs for the 45 different protocols, excluding those comparisons between identical protocols; (b) all permutation pairs for the 45 different protocols which share a common choice of expression measure; (c) all permutation pairs for the 45 different protocols which share a common choice of background subtraction excluding those comparisons between identical protocols; (d) all permutation pairs for the 45 different protocols which share a common choice of normalization excluding those comparisons between identical protocols.

Figure 2 shows a histogram of percent overlaps for the permutations of different protocols. The average overlap between gene lists from two calibration protocols is 76.0%. The distribution has four peaks, with the highest peak indicating comparisons between protocols sharing the same expression measure. The average overlap for gene lists produced from just Li-Wong protocols is 87.4 ± 3.4, for just RMA protocols is 88.9 ± 3.2 and for just MAS protocols is 88.8 ± 4.0. The lowest peak of the distribution is due to comparisons between protocols in which one uses MAS and the other uses RMA (compare with Figure 1). The next peak is from comparisons between RMA and Li-Wong, and the next peak (second highest) is from comparisons between Li-Wong and MAS.

For a given expression measure, the choice of background is more important than the choice of normalisation. Inspection of figure 1 shows that along the diagonal, collections of boxes of size 5 (due to the normalisation) all appear to have similar values. The average overlap in gene lists between protocols sharing the same expression measure and background subtraction is 92.7 ± 2.7 indicating that background information makes some improvement in consistency.

**Figure 3:**
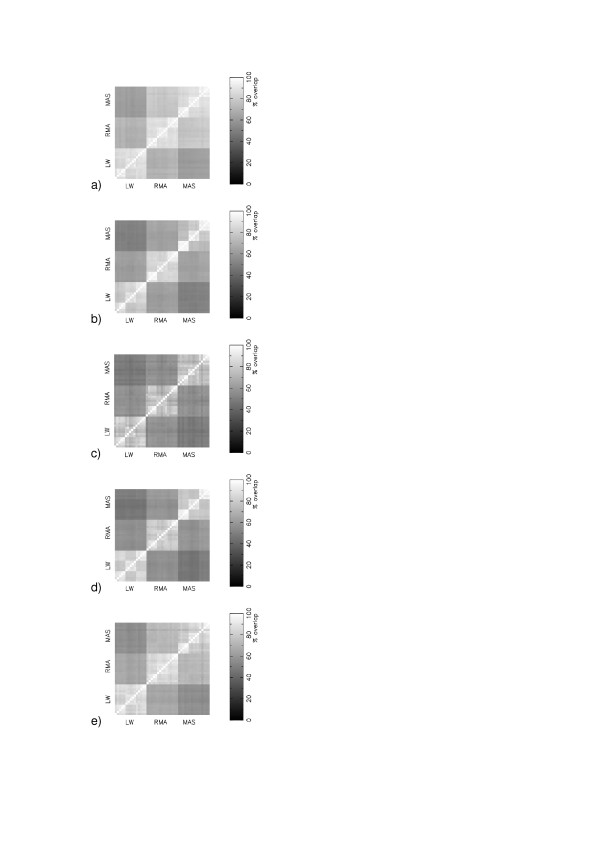
Matrices of consensus in the top 400 most significant changing genes (Z-score) in the 45 different protocols : (a) GSE1004; (b) GSE1703; (c) GSE1873; (d) GSE2401; (e) GSE2535.

Figure 3 shows matrices of comparisons for experiments GSE1004 [9], GSE1703 [13], GSE1873 [14], GSE2401 [15] and GSE2535 [16]. We find that differences in the expression measure is the dominant cause of disagreements in the gene lists between different protocols, irrespective of whether the experiment uses a human, rat or mouse chip, and irrespective of the number of biological replicates for each condition. If two calibration protocols share a common expression measure then the average percent overlap in their gene lists is 83% for GSE1703, 77% for GSE1873, 82% for GSE2401 and 85% for GSE2535.

**Figure 4:**
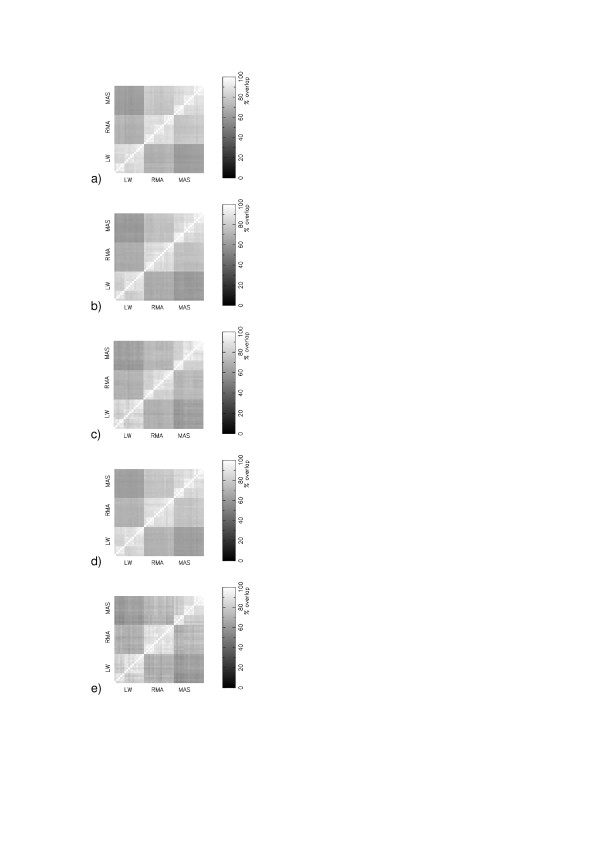
Matrices of consensus in the top 400 most significant changing genes (Z-score) in the 45 different protocols for GSE1004 using different chip definitions : (a) Affymetrix; (b) Unigene; (c) Refseq; (d) Entrez Gene; (e) Ensembl Transcript.

There exist collations of the information about transcripts and probes from different online resources [6]. Different probes from within a probeset may map to more than one transcript, and may be assigned erroneously to a gene to which they do not map. There are Chip Definition Files (CDFs) using mappings to Unigene, RefSeq, Entrez Gene and Ensemble Transcript amongst others [6]. Furthermore, Affymetrix provide a CDF produced from their original mapping. We have assessed whether the gene lists are strongly affect by the use of different probe mappings, through analysing GSE1004 using the 8^th ^build of the CDFs from [6] and the original Affymetrix CDF, Figure 4. Our results show that irrespective of which CDF is used, the dominant cause of discrepancy between different gene lists remains the choice of expression measure. If two calibration protocols share a common expression measure then the average percent overlap in their gene lists in the GSE1004 experiment is 88% for the Affymetrix CDF, 87% for the Ensembl Transcript CDF, 88% for the Entrez Gene CDF, 86% for the RefSeq CDF and 87% for the Unigene CDF.

**Figure 5:**
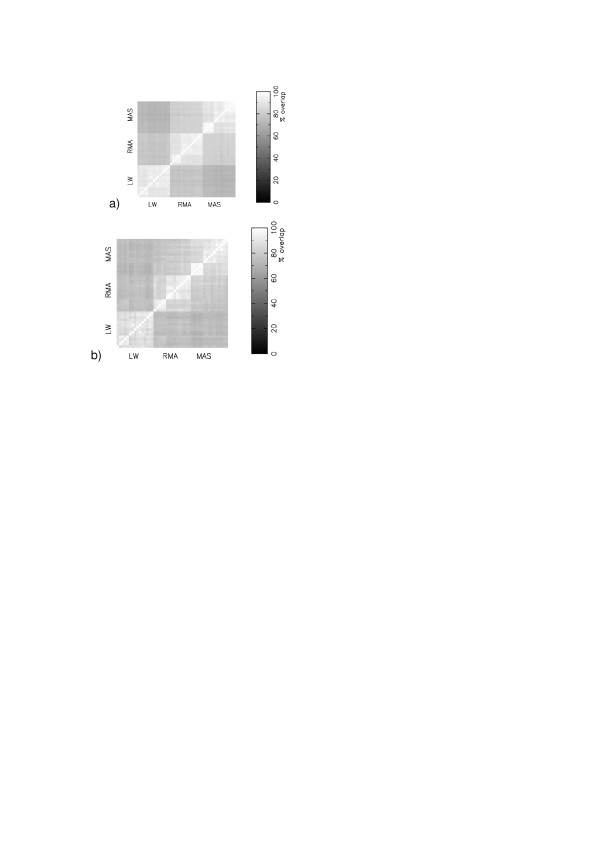
Matrices of consensus for those probesets which are all present in the GSE1004 experiment: (a) for the top 400 genes from each calibration protocol; (b) for the top 100 genes from each calibration protocol.

The MAS5 algorithm makes Present/Absent calls for each probeset. In order to assess whether these calls made a difference to our conclusions, we made a very conservative study of GSE1004, only sampling the genes which had been called present in all the chips. This was true for 2965 of the 12625 probesets. Figure 5 shows how the choice of expression measure is still the dominant cause of discrepancy between the gene lists for different protocols.

**Figure 6:**
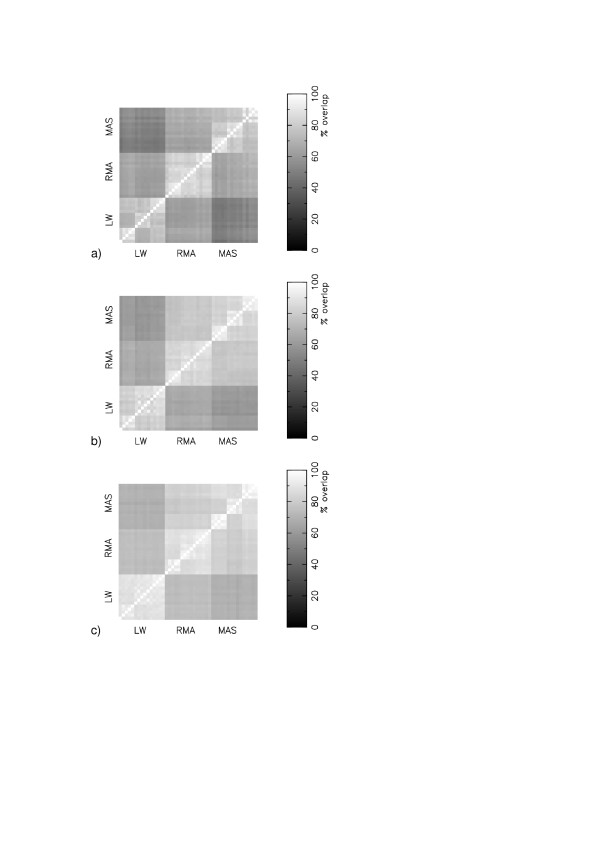
Matrices of consensus for the top 400 genes between different calibration protocols for GSE1004: (a) genes with small intensity; (b) genes with medium intensity; (c) genes with large intensity.

We wished to assess whether our findings are dependent upon the intensity of the genes. We broke the probesets for GSE1004 into three groups, those whose average intensity across the chips, when run with the standard RMA protocol, is either < 5.5 (4401 probesets), ≥ 5.5 and < 7 (3853 probesets) or ≥ 7 (4371 probesets). Figure 6 shows the genes with the highest intensity have the highest overlap between calibration protocols and those with the lowest intensity have the least overlap between calibration protocols. Irrespective of intensity, the choice of expression measure provides the biggest cause of discrepancy between gene lists produced from different calibration protocols.

**Figure 7:**
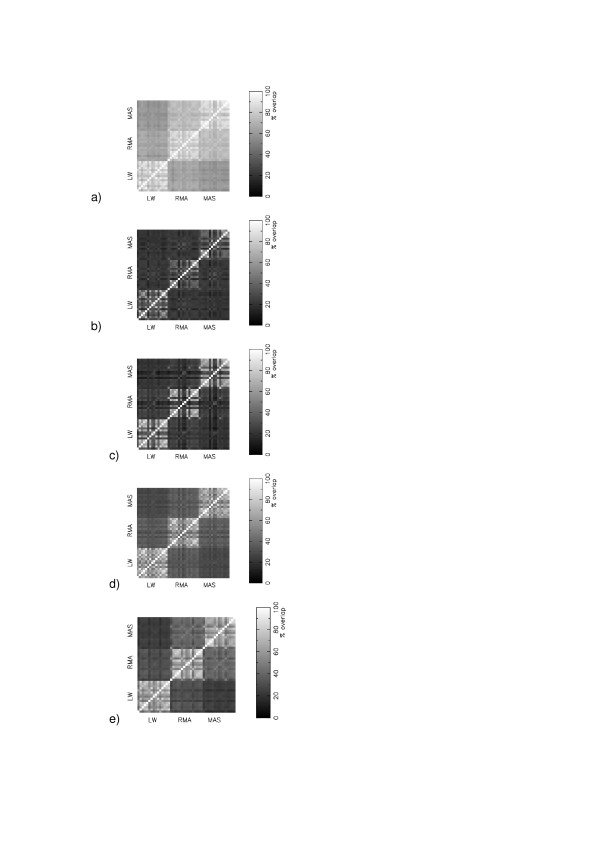
Matrices of consensus between different calibration protocols using t-tests to assess the 400 most significantly differentially expressed genes: (a) GSE1004; (b) GSE1703; (c) GSE1873; (d) GSE2401; (e) GSE2535.

We wished to determine whether our choice of Z-scores, rather than t-tests, was biasing our conclusions about the importance of expression measure. We calculated the most significantly changing genes for each of the five experiments, evaluated using the standard t-test formalism. Figure 7 shows that the typical discrepancy between calibration protocols using t-tests is much greater than found using the Z-score formalism. When an experiment contains only a few replicates, estimates of the mean and variance are noisy.

**Figure 8:**
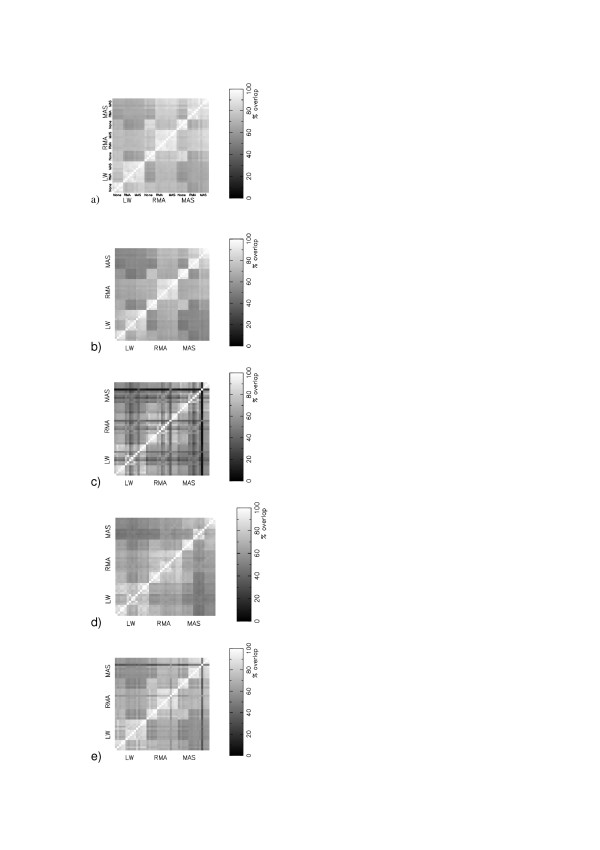
Matrices of consensus between different calibration protocols using fold change to assess the 400 most significantly differentially expressed genes: (a) GSE1004; (b) GSE1703; (c) GSE1873; (d) GSE2401; (e) GSE2535.

In order to confirm our hypothesis that Z-scores will provide a cleaner diagnostic than fold change, we calculated the largest fold changes for each of the five experiments. Figure 8 shows that for a given choice of background and expression measure, there is little difference in the gene lists for different normalisation choices. However, unlike the results for Z-scores, background choice and expression measure have a similar effect on the discrepancies between different calibration protocols.

## Discussion

Our results indicate that the calculation of the expression measure is the dominant cause of discrepancies between genelists produced from different calibration protocols. The expression measure calculation brings together data from multiple probes, and each of the probes may have a systematic error associated with it. Although great progress has been made, through the developments of MAS, MBEI and RMA, there are still inherent complications associated with calibrating Affymetrix data.

The MAS expression measure treats cross-hybridisation differently to RMA and Li-Wong and this difference will lead to discrepant estimates in the probe specific background. Ideally each probe would only map to a specific part of the sequence of interest, but in reality different mRNA fragments from various genes can stick to the same probes. In order to measure such cross-hybridisation Affymetrix introduced MisMatch probes to their chips. However, physical models of this process are now becoming available and it is now clear that a significant fraction of the MisMatch signal arises from hybridisation to transcripts with the PerfectMatch sequence [17]. One model of this process [17] ignores the order of the base composition, whereas another model of hybridization [18] explicitly accounts for the sequence order. Currently, there is no consensus on the dominant contributions to the hybridization physics, nor the most appropriate models in which to describe the effects. It is hoped that the increasingly detailed physical models of this process, [19], will shed light on this matter.

The Li-Wong and RMA expression measure used in this study both explicitly ignore cross-hybridisation and the only difference between the Li-Wong and RMA genelists arise from the two ways in which the model of a probe-set are constructed. RMA provides statistically robust averaging methods whereas Li-Wong fits a model that allows probe-specific binding affinities. Also, Li-Wong calculates expression on the original probe intensity values rather than the log-transformed ones used by RMA. However, the differences in gene lists between these different expression measures is still greater than 20% which shows the importance of the choice for a model and fitting paradigm. Our results agree with [2] in that we find from studying fold change (Figure 8), good agreement between methods that do not correct for background.

In line with other work, e.g. [20], we find that the lowest intensity genes result in a bigger discrepancy between calibration protocols, than those genes found with a larger intensity. Although our evidence from this study is weak, this is to be expected if the genes reported to be differentially expressed from the lowest intensity genes contain a larger number of false positives due to noise.

There is an ongoing debate over whether measures of differential expression based on either t-statistics [21] or fold-change [22] formalisms provide the most sensible way in which to generate gene lists. Microarray experiments typically contain small numbers of replicates, which means that any t-test formalism will attempt to calculate the mean and variance of a distribution from only a small number of values. The large numbers of probesets means that this approach will necessarily result in false positives when the numbers of replicates is small. Indeed, Figure 7 is consistent with an experiment in which two lists are compared, when each list contains a small number of common genes and a large set of random genes. We agree with [21], that the use of small sample sizes can lead to frustration amongst biologists chasing false leads. Our, somewhat brutual, advice to biologists is that such frustration is to be expected if your search is based around following genes identified by t-tests when you only have a small number of replicates.

We favour the choice of Z-score, rather than fold change, because we predict this should maintain the advantages of fold-change, namely reproducibility, as well as explicitly correcting for the non-linear response curves of microarray data. There are a number of expression measures that are affected by bias in the lowest and highest intensity ranges [2], and for each of these measures we recommend the use of Z-scores over fold change. However, the field of expression measure development is evolving and the work of Irizarry and colleagues is leading to measures in which the bulk of the biases are being removed. In the absence of intensity-dependence of ratio data Z-score and fold-change will result in the same ranking order of gene lists. Similarly, in the limit when the sliding window is set to 100% of the genes on the array, Z-score and fold change will result in the same ranking order. We hope that future work, based around following a similar approach to previous studies [23], also include an analysis of Z-scores in comparison to other statistical tests.

Irrespective of whether we use fold change or Z-score, our results agree with [2], in that we find the choice of normalization measure has little effect on the results compared to modifying the choice of background or expression measure. However, [5] suggested that normalization has a profound effect on differentially expressed genes from Affymetrix microarrays. The analysis of [5] used three statistical tests, namely parametric ANOVA, nonparametric ANOVA and SAM [24], each of which uses the small number of replicates for any gene to estimate statistical significance. Whereas, we have used Z-scores, which ignores the calculation to estimate variance in one gene, and instead only deals with its mean values in each condition, and estimates statistical significance based on comparing this difference with a population of hundreds of measurements of differential expression for different genes. We believe this approach is safer because there are usually not enough replicates to reliably estimate both the mean and variance of a single gene in standard microarray experiments.

The MAS5 algorithm [11] provides present/absent calls. We find that including only genes found to be present in every single chip provides a slight increase in the overlap between different calibration protocols.

Affymetrix annotate their GeneChips by aligning the probes to databases of genomic and transcript sequences [25]. If at least 9 of the 11 probes in a probe set match perfectly to a transcript, the assignment is considered a high quality assignment and is referred to by Affymetrix as a Matching Probes assignment (grade A). Affymetrix (2004) claim that over 70 percent of the probesets on the latest Human and Mouse GeneChips have grade A annotations. However, this leaves almost 30 percent of probe sets which contain three or more probes that do not align to transcripts for which they were designed. Moreover, the latest rat chip has only one third of its probe sets classified as A. Furthermore, on the older releases of GeneChips, there is much greater use of EST information, and hence more likely sources of errors. Thus many probes on GeneChips will not align to the transcripts to which they are assigned and there is a need to identify and remove such spurious probes [7]. Another complication is that some probes are found to match to more than one place in the genome, and so are particularly prone to cross-hybridisation. Using the Dai *et al*. CDFs tailored to specific annotation resources [6], we show that, irrespective of the annotation source, the biggest cause of discrepancy between gene lists from different calibration protocols is the choice of expression measure.

## Conclusion

We have systematically examined permutations of different calibration protocols for the analysis of Affymetrix GeneChips. Our results show that the lists of significant genes identified by sliding Z-scores show a clear demarcation between the different expression summaries. The overlap in significant genes for a given expression measure, chosen with either different backgrounds or different normalisations, is high, typically greater than 80%. These findings apply irrespective of which annotation resource has been used to assign probe mappings. Our results highlight that the major uncertainty in the calibration of Affymetrix microarray data is the choice of method in transforming the multiple probe intensities into one measure of expression.

## Methods

### 1) Affymetrix Chips used in the analysis

The GeneChip data in this study was extracted from the Gene Expression Omnibus database [26]. We extracted the data from 5 experiments, which were given the GEO IDs GSE1004, GSE1703, GSE1873, GSE2401 and GSE2535. Our sample includes data from Human, Rat and Mouse, chosen so we that we can assess whether our conclusions are dependent upon the species. We also sampled three experiments using HGU95Av2, chosen to see if our conclusions alter for different data taken from within a species.

a) GSE1004 [9] is a study of Duchenne Muscular Dystrophy (DMD) using Human HG-U95Av2 GeneChips. mRNA taken from twelve quadriceps biopsies from DMD patients was compared with twelve quadriceps biopsies from unaffected controls. Analysis of the GEO data indicates that one of the control chips cannot be analysed in the same batch as the others, meaning that our analysis compares twelve DMD chips versus eleven control chips.

b) GSE1703 [13] is a study of transcripts undergoing nonsense mediated decay (NMD) using Human HG-U95Av2 GeneChips. Two chips had mRNA from cells with RENT1 knocked down through siRNA, and two chips had mRNA from control cells.

c) GSE1873 [14] is a study of chronic intermittent hypoxia in obese mice using Mouse MOE430A 2.0 GeneChips. mRNA taken from five mice exposed to intermittent hypoxia was compared with five mice exposed to intermittent room air.

d) GSE2401 [15] is a study of the regulation of blood pressure in rat using Rat RAE230A GeneChips. mRNA taken from rats having acute hypotension induced by bleeding using ventricular punction was compared with control rats undergoing ventricular punction without bleeding. Four chips were made using the hypotension rats and five were made using the controls.

e) GSE2535 [16] is a study of gene expression changes induced by Imatinib using Human HG-U95Av2 GeneChips. mRNA was taken from diagnosed patients with chronic myeloid leukaemia, from responders and nonresponders to Imatinib, and from patients from either Leipzig or Mannheim. For our analysis we separated the data of Crossman into just two groups, 16 chips for responders and 12 for nonresponders

### 2) Calibration options

A welcome development in the field of microarray analysis has been the Bioconductor project [27]. Bioconductor aims to develop open-source tools (primarily in the language R) for the analysis of genomic data. Many of the statisticians researching numerical methods to deal with microarray data are using R and making their algorithms freely available through Bioconductor. The project is able to provide sophisticated, up to date tools for microarray analysis. Furthermore the project has a commitment to providing documentation and support for the tools it provides. Moreover, the associated newsgroup allows novice users to communicate directly with algorithm developers as well as experienced users.

Several of the Bioconductor software libraries are dedicated to the analysis of data from Affymetrix chips. The package "*affy*" allows the development of customised calibration protocols. It allows, through the use of the routine "*expresso*", choices from various background correction methods, normalisation routines and expression summaries (Table 1). Combining the three options for Background correction, five for normalisation and three for expression measure allows 45 possible calibration protocols.

### 2. i) Background correcting with affy

A proportion of an intensity measurement is not due to binding of the target to the probe. Even blank areas on the chips show some fluorescence and there will also be some non-specific binding contributing to the intensity measurement of a perfect match probe. Background correction is a contentious issue and so affy allows a choice from three.

One option is to simply ignore the background correction step altogether. This choice acknowledges that current background corrections may not successfully remove the background without increasing the noise level – the result of subtracting one noisy value from another noisy value is more noisy than either of the original values. A second option is that used by Affymetrix MAS 5.0 [11]. This involves using areas of the chip with the lowest fluorescence as an estimate of background. It also uses the information contained within the MM probes to estimate the levels of cross-hybridisation. A further choice [10] which estimates the background based on fitting a model to the distribution of intensities on the chip.

### 2. ii) Normalisation options with affy

Arrays can be normalised by making the average intensity on each array take the same average value. This "constant" correction is either the average intensity of a chosen baseline array or an arbitrary target intensity. A number of variations on this theme are possible. For example, scaling to housekeeping genes, using spiked-in mRNA's of known concentration, or using a subset of the data with the least variation between arrays. The Affy library allows the user to choose "invariant set" as a normalisation option.

A single scale factor, applied to all values, makes the assumption that the array effect is constant for all intensities. This does not appear to be the case for all microarray data and suggests the need for non-linear normalisation. Loess normalisation is a popular approach for dealing with non-linear scaling. A curve is fitted to the fold changes within a small intensity window. The values are then scaled such that the average fold change becomes zero within the window.

An alternative way to apply non-linear normalisation is to use quantiles [28]. This method forces the intensity distributions on each chip to be identical by ranking the intensities, and resetting the intensity values at each rank across all chips to be the mean of the intensities at that rank. Each gene is given a normalised intensity value that reflects its level of expression compared to all the other genes on the array. Q-Spline [29] normalisation functions like quantile normalisation, by forcing the intensities on each chip to have the same distribution. However, Q-Spline fits smoothing splines to the array and the target array quantiles. These splines are then used as signal dependent normalisation functions on the signals of the array. In practise, quantile and Q-spline normalisation produce very similar results.

Affy, via expresso, allows a choice from any of these five normalisations: constant; invariant set; loess; qspline; quantile.

### 2. iii) Calculating an Expression Measure with affy

A single Affymetrix GeneChip contains hundreds of thousands of different oligonucleotides, each present in millions of copies. To identify a single gene transcript, upto 20 probe pairs are used. Each probe pair consists of a perfect match (PM) probe and a mis-match (MM) probe. The MM is identical to the PM probe except for a single substitution at the central position, and is designed to identify non-specific hybridisation. Assuming a suitable normalisation and background subtraction have been performed, the multiple PM and MM values must be condensed into a single value as a measure of the expression of the transcript.

In early versions of Affymetrix's Microarray Analysis Suite (MAS), versions 4.0 and below, the expression measure used an "Average Difference" value: the mean of the PM-MM values for a given probeset. The motivation for this expression measure is simply that subtracting the MM value from the associated PM should remove the mismatch component of the PM intensity, and also the average over the adjusted PM values ought to result in an estimate of the mRNA concentration in the sample. Although widely used, Average Difference has found to be a poor measure of expression level [12]. A more robust measure called Signal has been introduced in MAS 5.0. Signal is based on a weighted average of the log of PM-MM [11].

An alternative philosophy for deriving an expression measure takes advantage of the fact that all Affymetrix chips of a given array design are created equal. This means that the response of a probe set will be constant from chip to chip. This allows a model to be made that measures both the probe response pattern and the expression of transcript on each chip [12]. Rather than taking each chip in isolation, as in Signal, the model based methods look at the responses across all the chips together. The model-based approach implemented in the analysis software d-chip is known as the Li and Wong method or the Model Based Expression Index (MBEI). An alternative model-based approach has been proposed [10] and is known as the Robust Multichip Average (RMA). The two methods use different models and different methods for parameter estimation. In this study we use Li-Wong models based only on the PM values.

### 3) We have used Z scores and T-tests as our measure of statistical significance of differential expression

Affymetrix data shows a non-linearity between signal intensity and transcript concentration. At the low and high ends of transcript concentration, a doubling in concentration results in a much lower change in fluorescent intensity [30]. Because fold changes measure differences in fluorescent intensity, they will therefore not be accurately measuring the differences in transcript concentration. Chemical saturation of GeneChips is likely responsible for the reduction in fold changes seen at high intensities [19]. Similar non-linear scanner curves in studies using cDNA glass-spotted arrays [31], suggest many of the physical processes underpinning the non-linearity may be common to a number of microarray technologies.

We believe that the reduction in fold changes seen at low intensities is likely due to cross-hybridisation between probes and the general population of mRNA. Because the population signal will be fairly constant between conditions, and contributes a significant fraction of the fluorescent intensity, the observed fold change will not be representative of the change in transcript abundance for the gene of interest. A Z-score derived from fold changes within a local intensity window corrects intensity dependent effects apparent in two-colour arrays [1]. Applying this methodology resulted in a list of significant genes that are evenly spread across the intensity range, following the distribution of the underlying population.

We have chosen to follow this. The Z-score, in this instance, is defined as the number of standard deviations a probesets fold change is away from the mean fold

Zprobeset=foldchangeprobeset−μpopulationσpopulation
 MathType@MTEF@5@5@+=feaafiart1ev1aaatCvAUfKttLearuWrP9MDH5MBPbIqV92AaeXatLxBI9gBaebbnrfifHhDYfgasaacH8akY=wiFfYdH8Gipec8Eeeu0xXdbba9frFj0=OqFfea0dXdd9vqai=hGuQ8kuc9pgc9s8qqaq=dirpe0xb9q8qiLsFr0=vr0=vr0dc8meaabaqaciaacaGaaeqabaqabeGadaaakeaacqWGAbGwdaWgaaWcbaGaemiCaaNaemOCaiNaem4Ba8MaemOyaiMaemyzauMaem4CamNaemyzauMaemiDaqhabeaakiabg2da9maalaaabaGaemOzayMaem4Ba8MaemiBaWMaemizaqMaem4yamMaemiAaGMaemyyaeMaemOBa4Maem4zaCMaemyzau2aaSbaaSqaaiabdchaWjabdkhaYjabd+gaVjabdkgaIjabdwgaLjabdohaZjabdwgaLjabdsha0bqabaGccqGHsisliiGacqWF8oqBdaWgaaWcbaGaemiCaaNaem4Ba8MaemiCaaNaemyDauNaemiBaWMaemyyaeMaemiDaqNaemyAaKMaem4Ba8MaemOBa4gabeaaaOqaaiab=n8aZnaaBaaaleaacqWGWbaCcqWGVbWBcqWGWbaCcqWG1bqDcqWGSbaBcqWGHbqycqWG0baDcqWGPbqAcqWGVbWBcqWGUbGBaeqaaaaaaaa@73A5@

change (equation 1). The standard deviation, σ, and mean fold change, μ, are derived from the population of genes with similar intensities to the gene of interest. In our analysis we used a sliding window of 1%.

We have also produced gene lists for each experiment using the standard t-test calculation. As we illustrate, the t-tests lists are very sensitive to the choice of calibration protocol.
